# Supplementary material for: ReportAGE: Automatically extracting the exact age of Twitter users based on self-reports in tweets
Source: PLoS One. 2022 Jan 25;17(1):e0262087. doi: 10.1371/journal.pone.0262087 (PMC8789116; doi:10.1371/journal.pone.0262087)
Supplement: S1 Text — (PDF) [file pone.0262087.s001.pdf]

Identifying the Exact Age of Twitter Users from Self-Reports in Tweets: Annotation Guidelines

Department of Biostatistics, Epidemiology, and Informatics
Perelman School of Medicine
University of Pennsylvania

Last updated: July 30, 2020

The following guidelines will help annotators distinguish tweets that self-report the user's exact age ("age") from tweets that do not ("no age"). Annotators should label tweets as "age" or "no age" based only on the information provided by the tweets and posting dates; they should not consider external information provided by the user's profile or by clicking on URLs within the tweet.

Tweets should be annotated as "age" if the user's *exact* age can be determined at the time the tweet was posted, as in:

(1) `I know I'm a pregnant 34-year-old but I genuinely want this for Xmas. [URL]`
(2) `It's my 21st birthday today. But who cares..... ITS FINALLY AUGUST!!!!!!!! That's what really matters` 😭😭😍😍😍💖💖💖💖💖

In Tweets (1) and (2), the users explicitly state their exact age: 34 and 21, respectively. In other tweets, however, the user's age may be more implicit, as in:

(3) `This is the most beautiful cake I've ever had! Thank you @JAntal I love it!!! #GoldenBirthday #blessed`

Although Tweet (3) does not explicitly state an age, the user's exact age can be determined from *#GoldenBirthday* and the posting date. A "golden birthday" refers to the birthday when one turns the same age as the day on which he or she was born. This tweet was posted on August 22, 2013, so the user was 22 when the tweet was posted.

Similarly, some tweets may state an age, but that age may not explicitly indicate the exact age of the user at the time the tweet was posted, as in:

(4) `Still shocks me when Spencer casually talks about his 21st bday that was 3 years ago and that's how long UNTIL my 21st.` 😂
(5) `It's crazy, tomorrow I'll be 20. I'm getting so OLD.` 🤦‍♀️

Tweets (4) and (5) should be annotated as "age" because, although the users do not explicitly state their exact age, it still can be determined. Tweet (4) indicates that the user will be 21 years old in 3 years, so the user was 18 when the tweet was posted. Tweet (5) indicates that the user will be 20 tomorrow, so she was 19 when the tweet was posted.

Whereas Tweets (4) and (5) specify when the users will be the age mentioned in their tweets, other tweets may refer to a future age more generally, as in:

(6) `can't believe im going to be 21 .... i actually want to be a teenager again with no responsibilities` 😒
(7) `Planning a HUGE party for my 25th birthday...`

Although Tweets (6) and (7) do not specify when the users will be 21 and 25, respectively, annotators may reasonably assume that the users will be those ages on their *next* birthday, so the tweets should be annotated as "age."

However, tweets should be annotated as "no age" if they are ambiguous about whether an age refers to the future or the present, as in:

(8) `I graduate in May only focusing on me and my child.. watch me at 21` 😚

Tweet (8) is ambiguous about whether the user was 21 years old when the tweet was posted, or whether the user was referring to a future age.

Tweets should also be annotated as "no age" if they are ambiguous about whether an age refers to the past or the present, as in:

(9) `Had just turned 18 then found out I was pregnant 2 weeks later [URL]`

Tweet (9) is ambiguous about whether the user was still 18 years old when she posted the tweet, or whether she is referring to an age further in the past.

Tweets that refer to an age in the past should be annotated as "age" only if they indicate when the user was that age, as in:

(10) `Thanks for celebrating my 21st over the weekend, bestie.` 💓🎉
(11) `Had a great night celebrating with the other birthday girl!! We're like red wine.. Only getting better with age! #23`

Tweets (10) and (11) should be annotated as "age" because they indicate that the users turned 21 and 23, respectively, very recently.

Tweets should be annotated as "no age" if they mention a number that clearly does not refer to the user's age; however, if the tweet plausibly could be indicating the user's exact age and does not provide enough evidence otherwise, it should be labeled as "age," as in:

(12) `@KYOnePercenter I'm 17, but good try`

Similarly, tweets should be labeled as "no age" if they mention an age that clearly does not refer to the user; however, if the tweet plausibly could be referring to the user and does not provide enough evidence otherwise, it should be labeled as "age," as in:

(13) #Adulting is being 28 years old,eating French Toast Crunch cereal, watching a VHS of Saturday AM cartoons, w/ a color changing Aladdin spoon
